# Community at the Centre of Future Food Systems

**DOI:** 10.3390/nu14234951

**Published:** 2022-11-22

**Authors:** Colin Bell, Penelope Love

**Affiliations:** 1 Institute for Health Transformation, School of Medicine, Deakin University, Geelong, VIC 3216, Australia; 2 Institute for Physical Activity and Nutrition, School of Exercise and Nutrition Sciences, Deakin University, Geelong, VIC 3216, Australia

Have you thought about what it is you love about food? It is likely that it is not as much about the nutrients in the food or even how tasty the food is as it is about the circumstances in which your food was sourced, prepared, and eaten, and the connections you have with the people involved.

Indigenous knowledge systems teach us that connections with nature and culture are central to our identity and that food is central to those connections [1]. Food connects us to the land, oceans and sky that sustain us, as well as to our cultures. Food helps us know where we are from, ‘who we are’ and to ‘feel good about ourselves’ [2].

Community is the level of society where food-related connections to people and place occur. As such, community needs to be at the centre of future food systems in the same way food markets are at the centre of our villages, towns and cities. Increased interaction between farmers and consumers may help to achieve more environmentally sustainable food supply chains [3]. It may also reduce the stranglehold that global food manufacturers have on food supply and decrease the supply of ultra-processed foods [4]. Community, school and home gardens will improve food literacy and accessibility, and the affordability of healthy foods [5]. Where people are separated from community and culture due to climate change, conflict or economic opportunities, food can help bring them back together [6].

Globally, countries are pushing back on corporate and climate change disruptions to food systems and are looking for ways to strengthen community involvement in food systems, so that healthy and environmentally friendly food choices are easier to make. To achieve this, policymakers need access to the latest evidence on evolving dietary patterns and on effective solutions, and this Special Issue on Progress in Community Nutrition: Dietary Patterns and Planetary Health provides some of this evidence.

Using 2015 data from over 20,000 Canadians, Shafiee et al. identified cooked regular lean or extra-lean ground beef and white rice as the most frequently consumed animal-based and plant-based foods, respectively [7]. Based on a literature review, Gibbs and Cuppuccio identify meat appreciation, health concerns, convenience, and expense as barriers to transitioning to more plant-based food systems, which have a smaller environmental footprint than animal-based food systems [8]. Using unique data from the School Fruit and Vegetable Scheme in European Union Member States, Comino et al. found that over a 7-year period (2009/10 to 2016/17), it was possible for school children to receive up to 20 kg of fruit or vegetables delivered over 150 days [9]. However, not every child targeted by the scheme ended up with fruit or vegetables in their hands, and delivery days and the amount of fruit and vegetables provided varied considerably across countries. These studies illustrate the challenges faced by communities to transition to more environmentally friendly dietary patterns.

With the development of future dietary guidelines for Australia in mind, Wingrove et al. [10] point to the need for the inclusion of evidence on environmental sustainability and equity, in addition to evidence on dietary exposure and health. Research from Japan, led by Kurisaki and Kushida [11], and from Vietnam, led by Thien Mai [12], highlight how difficult dietary exposures are to measure, and of the need for quality and validated measurement techniques. The contribution from Du Plessis and colleagues reminds us that the translation of dietary evidence and guidelines into messages suitable for different target audiences is as important as the evidence itself [13]. Lastly, Swart et al., with their unique study on the dietary intake and diversity of landfill waste-pickers in South Africa, urges us not to neglect the nutritional status of vulnerable communities [14].

If community is more centrally engaged in the food system, it is possible that naturally occurring and culturally appropriate foods can be distributed more equitably, with an associated reduction in health disparities. Zoe et al. note in their scoping review that Chinese immigrants to Canada and the United States consumed less healthful diets (for example, higher caloric intakes and higher meat and alternative intakes) with greater acculturation [15]. Importantly, limited access to traditional or healthy foods was associated with acculturation. They note that ‘decisions regarding policies on immigration and neighbourhood planning can also consider the health benefits associated with areas of high immigrant density, improve access to ethnic foods, and promote sharing of cultural food practices’ [15]. Akbar and colleagues used a taro plant to illustrate themes that emerged from interviews with diverse representatives of Māori and Pasifika diaspora communities in Queensland, Australia [16]: ‘Identity, hospitality and reciprocity, and spirituality form the roots or foundation of the meaning of food for Māori and Pasifika peoples; solutions and barriers form the soil or structure from which these understandings and practices of food are nourished or restricted and; physical and mental health, expectations and obligations, and stigma and shame form the leaves of the taro plant, representing the surface-level experiences and challenges of food insecurity’ [16]. The authors conclude that ‘when foods (including cultural foods) are not available or accessible, individuals experience not only a loss of physical health but lose connections to their broader identity and their link to their ancestral homes’ [16].

The EAT–Lancet Commission framework for identifying ‘win-win’ diets (healthy and environmentally sustainable) was proposed as ‘universal for all food cultures and production systems in the world, with a high potential of local adaptation and scalability’ [17]. This Special Issue reveals that such local adaption is best achieved by positioning the community at the centre of future food systems.
